# Self-Medication Safety Behaviours and Recognition of Pharmacological Risks: A Clinical Pharmacology Perspective

**DOI:** 10.7759/cureus.117147

**Published:** 2026-09-29

**Authors:** Eszter-Anna Dho-Nagy, Erika Gyöngyi Bán, Attila Brassai

**Affiliations:** 1 Department of Pharmacology, George Emil Palade University of Medicine, Pharmacy, Science and Technology of Târgu Mureș, Târgu Mureș, ROU; 2 Doctoral School of Medicine and Pharmacy, George Emil Palade University of Medicine, Pharmacy, Science and Technology of Târgu Mureș, Târgu Mureș, ROU

**Keywords:** active-substance duplication, clinical pharmacology, drug interactions, medication literacy, medication safety, over-the-counter medicines, pharmacological risk, self-medication

## Abstract

Background: Safe self-medication with over-the-counter (OTC) medicines requires identification of active substances, recognition of clinically relevant interacting exposures, and awareness of when continued self-treatment is no longer appropriate. Whether self-reported safety behaviours correspond to recognition of the pharmacological risks they are intended to prevent remains insufficiently understood.

Objective: To examine respondent-level correspondence between self-reported OTC safety behaviours and recognition of conceptually related pharmacological risks among adults without healthcare training, and to translate the observed patterns into a clinical-pharmacology framework for safer self-medication.

Methods: An exploratory secondary analysis was performed using data from a Romanian cross-sectional study of OTC medication risk literacy. The analysis included 206 respondents without a healthcare background. Five conceptually matched knowledge-behaviour comparisons were examined across active-substance duplication, interaction-related risks, and the boundary of self-treatment. Associations were assessed using odds ratios (ORs), 95% confidence intervals (CIs), and two-sided Fisher’s exact tests, with Holm adjustment for multiple comparisons.

Results: Reported safety behaviour did not invariably correspond to recognition of the related pharmacological risk. Among 143 respondents reporting consistent active-ingredient checking, 22 (15.4%) failed to recognise duplicate paracetamol exposure; recognition was associated with ingredient checking (OR 2.95, 95% CI 1.48-5.88; p = 0.003). Among 143 respondents reporting routine interaction checking, discordance was 14 (9.8%) for the anticoagulant-related scenario, 25 (17.5%) for herbal products and dietary supplements, and 87 (60.8%) for antihistamine-alcohol use. Correspondence was greater for the boundary of self-treatment: only seven (4.3%) of 162 respondents reporting avoidance of prolonged OTC use despite persistent symptoms failed to recognise the need for professional evaluation. Following Holm correction, only the active-substance duplication association remained statistically significant.

Conclusions: Self-reported OTC safety behaviours should not be assumed to represent successful application of the underlying pharmacological reasoning. Correspondence was domain-dependent and appeared less consistent for risks requiring identification and interpretation of pharmacological exposures than for persistent symptoms requiring evaluation. These findings support making the pharmacological reasoning underlying OTC safety recommendations more explicit and informed the proposal of a clinical-pharmacology framework for future OTC safety communication.

## Introduction

Over-the-counter (OTC) medicines are an integral component of self-care, but their non-prescription status does not imply the absence of pharmacological risk. Inappropriate dosing, incorrect self-diagnosis, adverse effects, and drug interactions remain relevant safety concerns [1]. OTC medicine safety depends on the active substance, dose and duration of exposure, concomitant medicines and other substances, patient-specific characteristics, and the clinical context in which self-treatment occurs. European data further indicate that OTC use is influenced by demographic factors, chronic disease, pain, healthcare access, and concurrent medicine use [2]. Self-medication is common across Europe and is shaped by previous experience, habits, symptom interpretation, and patterns of healthcare use [3,4].

Medication literacy provides an important framework for understanding the safe use of OTC medicines and refers to an individual’s ability to obtain, comprehend, process, and use medication-related information to make informed decisions [5]. In self-medication, this includes recognising active-substance duplication, interactions, contraindications, inappropriate dose or duration, adverse effects, and circumstances requiring professional evaluation. However, access to medication information does not necessarily ensure correct interpretation or application of pharmacological risk [6-9]. Difficulties in understanding medication instructions may be influenced by terminology, numerical presentation, and the complexity of dosing information [10], while population studies have demonstrated gaps in dose calculation, recognition of contraindications, adverse effects, and interaction-related risks [11].

Knowledge and behaviour therefore represent related but distinct dimensions of medication safety. Individuals may report reading medicine information, checking active ingredients, or searching for interactions without necessarily recognising the corresponding pharmacological problem in a specific situation. Previous research has shown that OTC risk perception and information-seeking behaviour vary according to the medicine and perceived level of safety [12]. Similarly, although an association between OTC knowledge and reported practice has been described, this relationship is not necessarily strong enough to consider the two constructs interchangeable [13].

This distinction is particularly relevant because common recommendations for safe self-medication are often expressed as behavioural instructions, such as checking active ingredients, checking for interactions, following the recommended dose, or seeking medical advice when symptoms persist [6-9]. These behaviours are only meaningful if the individual can identify the pharmacological risk they are intended to prevent. For example, ingredient checking requires recognition of the same active substance across different products, whereas interaction checking requires consideration of prescription medicines, OTC products, alcohol, herbal products, and dietary supplements.

Our preceding cross-sectional study evaluated OTC medication risk literacy through several domains, including scenario-based risk recognition and self-reported safety behaviours. The original analysis examined these domains primarily separately and according to healthcare background. Although previous research has examined associations between OTC knowledge and reported practice [13], correspondence between individual safety behaviours and recognition of conceptually matched pharmacological risks has received limited attention.

Building on this work, the present exploratory secondary analysis examined correspondence between conceptually matched safety behaviours and pharmacological risk recognition among adults without healthcare training. We focused on active-substance duplication, interaction-related risks, and the boundary of self-treatment, namely recognition of circumstances in which self-treatment should stop and professional evaluation should be sought. A secondary objective was to interpret the observed patterns through a clinical pharmacology-informed framework, identifying reasoning steps that may support safer self-medication. The proposed checklist was not considered an empirical outcome of the present study, but rather a hypothesis-generating clinical-pharmacology framework developed from the interpretation of the observed knowledge-behaviour discrepancies.

## Materials and methods

Study design and population

The present study is an exploratory secondary analysis of data obtained from a cross-sectional observational study of OTC medication risk literacy conducted in Romania between June and July 2026. The parent study used an anonymous online questionnaire developed by the authors addressing demographic characteristics, scenario-based OTC risk recognition, confidence and self-perceived knowledge, self-reported medication-safety behaviours, information sources, medication exposure, and pharmacovigilance awareness. The complete questionnaire and its development and psychometric assessment procedures have been previously published in the Open Access parent study [14]. The present analysis was restricted to the 206 respondents classified as members of the general public without a healthcare background. This restriction was selected because the purpose of the present study was not to repeat the healthcare-background comparison performed in the parent analysis, but to examine the relationship between reported safety practices and scenario-based pharmacological risk recognition among individuals without formal healthcare training. Ethical approval, informed-consent procedures, and recruitment strategy were also previously reported in the parent study [14]. No data were missing for the variables included in the five knowledge-behaviour comparisons.

Selection of knowledge-behaviour comparisons

For this exploratory secondary analysis, five conceptually matched knowledge-behaviour comparisons were examined across three pharmacologically relevant domains. Pairings were selected when a scenario-based risk-recognition item and a self-reported behavioural item addressed the same or a closely corresponding medication-safety process. The five pairings were defined specifically for the present exploratory secondary analysis based on conceptual correspondence between the available questionnaire items and were not prespecified as paired constructs in the original study design.

Active-Substance Duplication

Recognition of simultaneous use of two paracetamol-containing products was compared with the reported practice of checking whether OTC medicines used during the same period contained the same active ingredient.

Interaction-Related Risks

Reported routine checking for potential interactions was examined separately against recognition of risks associated with (1) OTC analgesic use during anticoagulant treatment, (2) concomitant antihistamine and alcohol use, and (3) herbal products or dietary supplements. These scenarios were analysed separately because they represent distinct pharmacological contexts.

Boundary of Self-Treatment

Recognition that persistent symptoms despite prolonged OTC use should prompt professional evaluation was compared with reported avoidance of prolonged OTC use when symptoms persisted.

Classification of reported safety behaviour

For positively framed safety behaviours, responses of Always or Frequently were classified as consistent safety behaviour. For the negatively framed item concerning prolonged OTC use despite persistent symptoms, responses of Never or Rarely were classified as consistent safety behaviour.

For each knowledge-behaviour comparison, respondents were classified into four categories: (1) correct risk recognition with consistent safety behaviour; (2) incorrect risk recognition despite consistent safety behaviour; (3) correct risk recognition without consistent safety behaviour; and (4) incorrect risk recognition without consistent safety behaviour.

Particular attention was given to the second category, representing respondents who reported performing the recommended safety behaviour but did not correctly recognise the corresponding pharmacological risk. This pattern was termed behaviour-recognition discordance for the purposes of the present analysis.

Statistical analysis

Categorical variables were summarised as counts and percentages. For each of the five conceptually matched knowledge-behaviour comparisons, 2 × 2 contingency tables were constructed. Associations were expressed as odds ratios (ORs) with 95% confidence intervals (CIs) and assessed using two-sided Fisher’s exact tests because of small cell counts in several comparisons. Given the exploratory nature of the analysis, unadjusted p-values are presented alongside Holm-adjusted p-values to account for multiple testing across the five principal comparisons.

To assess the robustness of the behavioural classification, a sensitivity analysis applied a more restrictive definition of consistent safety behaviour: Always for positively framed items and Never for the negatively framed item concerning prolonged OTC use despite persistent symptoms.

There were no missing values for the variables included in these analyses; therefore, no imputation was required. Data were managed using Microsoft Excel (Microsoft Corp., Redmond, WA, USA), and statistical analyses were performed using GraphPad Prism version 7 (GraphPad Software, La Jolla, CA, USA). A two-sided p < 0.05 was considered statistically significant.

## Results

Overview of knowledge-behaviour correspondence

Five conceptually matched knowledge-behaviour comparisons were examined among 206 respondents without healthcare training. Complete 2 × 2 contingency table results, ORs with 95% CIs, two-sided Fisher’s exact test p-values, and Holm-adjusted p-values are presented in Table 1.

**Table 1:** Pharmacological risk recognition according to corresponding self-reported safety behaviour among respondents without a healthcare background (N = 206) Consistent safety behaviour was defined as “Always” or “Frequently” for positively framed behaviours and “Never” or “Rarely” for avoidance of prolonged OTC use despite persistent symptoms. “Other behaviour” comprised all remaining response categories. Percentages are calculated within the corresponding behavioural group. Odds ratios compare the odds of correct pharmacological risk recognition among respondents reporting consistent safety behaviour with those reporting other behaviour. All comparisons were assessed using two-sided Fisher’s exact tests. Holm adjustment was applied across the five principal comparisons. OTC: over-the-counter

Knowledge-behaviour comparison	Consistent safety behaviour, n	Correct recognition, n (%)	Incorrect recognition, n (%)	Other behaviour, n	Correct recognition, n (%)	Incorrect recognition, n (%)	OR (95% CI)	Fisher’s exact p	Holm-adjusted p
Paracetamol duplication/active-ingredient checking	143	121 (84.6)	22 (15.4)	63	41 (65.1)	22 (34.9)	2.95 (1.48-5.88)	0.003	0.014
Anticoagulant-related risk/interaction checking	143	129 (90.2)	14 (9.8)	63	51 (81.0)	12 (19.0)	2.17 (0.94-5.00)	0.072	0.145
Antihistamine-alcohol risk/interaction checking	143	56 (39.2)	87 (60.8)	63	20 (31.7)	43 (68.3)	1.38 (0.74-2.59)	0.349	0.349
Herbal product/dietary supplement risk/interaction checking	143	118 (82.5)	25 (17.5)	63	43 (68.3)	20 (31.7)	2.20 (1.11-4.35)	0.028	0.113
Persistent symptoms/avoidance of prolonged OTC use	162	155 (95.7)	7 (4.3)	44	38 (86.4)	6 (13.6)	3.50 (1.11-11.01)	0.035	0.113

Among respondents classified as consistently performing the corresponding safety behaviour, the proportion who nevertheless failed to recognise the matched pharmacological risk varied substantially across domains, ranging from seven (4.3%) of 162 respondents for persistent symptoms to 87 (60.8%) of 143 respondents for concomitant antihistamine and alcohol use.

Active-substance duplication

A total of 143 (69.4%) respondents reported consistent active-ingredient checking. Among these respondents, 121 (84.6%) correctly recognised the paracetamol-duplication risk, whereas 22 (15.4%) did not (Table 1). Among the remaining 63 (30.6%) respondents who did not report consistent active-ingredient checking, 41 (65.1%) correctly recognised the risk, while 22 (34.9%) did not.

Consistent active-ingredient checking was associated with higher odds of correct recognition of duplicate paracetamol exposure (OR 2.95, 95% CI 1.48-5.88; two-sided Fisher’s exact p = 0.003; Holm-adjusted p = 0.014).

Interaction-related risk recognition

Among the 206 respondents, 143 (69.4%) reported Always or Frequently checking whether an OTC medicine could interact with other medicines or products. Risk recognition among these respondents varied according to the pharmacological context (Table 1).

Anticoagulant-related risk

Among respondents reporting consistent interaction checking, 129 (90.2%) correctly recognised the risk associated with OTC analgesic use during anticoagulant treatment, whereas 14 (9.8%) did not. Among the 63 respondents reporting other interaction-checking behaviour, 51 (81.0%) correctly recognised the risk, whereas 12 (19.0%) did not. Consistent interaction checking was not significantly associated with correct recognition of the anticoagulant-related scenario (OR 2.17, 95% CI 0.94-5.00; two-sided Fisher’s exact p = 0.072; Holm-adjusted p = 0.145).

Antihistamine and alcohol

Among respondents reporting consistent interaction checking, 56 of 143 (39.2%) selected the predefined safest response to concomitant antihistamine and alcohol use, whereas 87 (60.8%) of 143 did not. Among the 63 respondents reporting other interaction-checking behaviour, 20 (31.7%) selected the predefined safest response, whereas 43 (68.3%) did not. Consistent interaction checking was not significantly associated with correct recognition of this scenario (OR 1.38, 95% CI 0.74-2.59; two-sided Fisher’s exact p = 0.349; Holm-adjusted p = 0.349). Overall, 76 (36.9%) of 206 respondents selected the predefined safest response.

Herbal products and dietary supplements

Among respondents reporting consistent interaction checking, 118 (82.5%) of 143 correctly recognised that herbal products and dietary supplements may cause adverse effects or interact with medicines, whereas 25 (17.5%) did not. Among respondents reporting other interaction-checking behaviour, 43 of 63 (68.3%) correctly recognised this risk, whereas 20 (31.7%) did not. Consistent interaction checking was associated with higher odds of correct recognition in the unadjusted analysis (OR 2.20, 95% CI 1.11-4.35; two-sided Fisher’s exact p = 0.028), but this association did not remain statistically significant after Holm adjustment (adjusted p = 0.113).

Boundary of self-treatment

Among the 206 respondents, 162 (78.6%) reported Never or Rarely continuing an OTC medicine for a prolonged period when symptoms persisted. Of these, 155 (95.7%) correctly recognised the corresponding need for professional evaluation, whereas seven (4.3%) did not (Table 1).

Among the 44 respondents (21.4%) who did not consistently report avoiding prolonged self-treatment, 38 (86.4%) correctly recognised the need for professional evaluation, whereas six (13.6%) did not. Consistent avoidance of prolonged self-treatment was associated with higher odds of correct risk recognition in the unadjusted analysis (OR 3.50, 95% CI 1.11-11.01; two-sided Fisher’s exact p = 0.035), but this association did not remain statistically significant after Holm adjustment (adjusted p = 0.113).

Adjustment for multiple comparisons

Holm adjustment was applied across the five principal knowledge-behaviour comparisons. Following adjustment, only the association between consistent ingredient checking and correct recognition of duplicate paracetamol exposure remained statistically significant (Holm-adjusted p = 0.014). The associations for herbal products and dietary supplements (adjusted p = 0.113), persistent symptoms (adjusted p = 0.113), anticoagulant-related risk (adjusted p = 0.145), and antihistamine-alcohol use (adjusted p = 0.349) were not statistically significant after adjustment.

## Discussion

Principal findings

Previous European and Romanian research has identified the widespread use of self-medication, the central role of community pharmacists in supporting its safe use, and persistent gaps between the medication-safety information offered and the information understood or retained by consumers [3,6-8,15,16]. The present analysis extends these observations by examining whether reported safety behaviours correspond to recognition of the specific pharmacological risks they are intended to prevent.

Correspondence varied across the investigated domains. The largest observed association concerned active-substance duplication, although 15.4% of respondents reporting ingredient checking still failed to recognise duplicate paracetamol exposure. This was also the only association that remained statistically significant after Holm adjustment. Among respondents reporting routine interaction checking, discordance ranged from 9.8% for anticoagulant-related risk to 60.8% for antihistamine-alcohol use, whereas only 4.3% of those reporting avoidance of prolonged self-treatment failed to recognise the need for professional evaluation. These findings suggest that generic safety behaviours such as checking ingredients or checking interactions may not fully capture the pharmacological reasoning required for safe self-medication, supporting the development of a more explicit clinical-pharmacology checklist.

Active-substance duplication

Active-substance duplication showed the largest observed OR among the five comparisons, but 15.4% of respondents reporting routine ingredient checking still failed to recognise duplicate paracetamol exposure. This finding is consistent with evidence that consumers may not reliably identify the same active substance across multiple non-prescription products. In a structured study involving 500 adults, 45.6% demonstrated potential overdose by taking two acetaminophen-containing products within a 24-hour period, indicating that active-ingredient misunderstanding can translate into clinically important duplicate exposure [17]. The present results extend this concern by showing that reported ingredient-checking behaviour and successful recognition of duplicate exposure were associated but not equivalent. From a clinical-pharmacology perspective, this distinction is important: medication safety depends on the active substance and total exposure, rather than on each commercial product considered separately. Thus, following the recommended dose of individual products does not necessarily prevent excessive cumulative exposure when the same active substance is present in more than one preparation.

Interaction-related risks

Interaction checking showed considerably more heterogeneous correspondence with pharmacological risk recognition. A Swiss community-pharmacy study similarly showed that interaction risk related to self-medication may not be fully recognised by medicine users. Among regular pharmacy customers, 15.9% had a potential interaction between an OTC product used for self-medication and prescribed treatment, whereas only 46.8% were aware that such interactions could occur [18]. Despite almost 70% of respondents reporting routine interaction checking, discordance varied substantially according to the exposure considered. Previous studies have also demonstrated incomplete patient awareness of prescription-OTC interactions and limited recognition of herb- and supplement-drug interactions. These findings suggest that the instruction to check interactions may be pharmacologically underspecified: interaction assessment requires identification of all relevant concomitant exposures, including prescription and OTC medicines, alcohol, herbal products, and dietary supplements [19].

The particularly high discordance for antihistamine-alcohol use should be interpreted cautiously because the original scenario did not distinguish antihistamines according to sedative potential. Nevertheless, the overall pattern supports explicitly defining the interaction context, rather than assuming that consumers interpret all potential interacting exposures as equivalent.

Boundary of self-treatment

The boundary of self-treatment showed a different pattern, with only 4.3% discordance among respondents reporting avoidance of prolonged OTC use. Although the association did not remain statistically significant after multiplicity adjustment, the descriptive finding suggests that recognising persistent symptoms as an indication for professional evaluation may be more intuitive than identifying duplicate exposure or integrating heterogeneous interaction risks.

From a clinical-pharmacology perspective, duration of self-treatment should not be considered independently of therapeutic response. Persistence or worsening of symptoms changes the benefit-risk assessment of continued exposure and should prompt reconsideration of whether self-medication remains appropriate. This provides the clinical basis for defining an explicit stop-or-escalate boundary within OTC medication safety. Community-pharmacy research indicates that counselling and referral decisions in self-medication require structured assessment of symptoms, medicine use, contraindications, and circumstances in which self-care is no longer appropriate [7-9].

Clinical-pharmacology translation: a proposed OTC self-medication checklist

The observed knowledge-behaviour discordance suggests that broad medication-safety instructions may be insufficient when the specific pharmacological task underlying the recommended behaviour is not made explicit. Advice to “check the ingredients,” for example, may not ensure recognition of the same active substance across differently branded products or of the cumulative exposure arising from their concurrent use. Similarly, advice to “check for interactions” may not prompt consideration of prescription medicines, other OTC products, alcohol, herbal medicines, or dietary supplements. This distinction is pharmacologically important because interaction risk may arise from both pharmacokinetic and pharmacodynamic mechanisms, and because relevant exposures may include non-prescription medicines, prescription treatment, food supplements, and herbal products [20-22].

We therefore propose a concise clinical-pharmacology checklist that translates medication-risk assessment into six practical decision points as shown in Table 2. Three components are directly informed by the present findings: identification of active substances, consideration of interaction contexts, and recognition of the point at which self-treatment should stop and professional advice should be sought. Total exposure, patient-specific susceptibility, and therapeutic response are clinically informed extensions of these findings. The checklist is therefore data-informed rather than wholly data-derived and should be regarded as a proposed educational and counselling framework, rather than a validated decision-support instrument.

**Table 2:** Proposed clinical-pharmacology checklist for OTC self-medication OTC: over-the-counter The table was developed by the authors based on the findings of the present study.

Number	Clinical-pharmacology step	Patient-oriented question	Pharmacological purpose
1	Identify the active substance	What active substance or substances does each product contain?	Enables recognition of identical active substances across differently branded or combination products.
2	Consider total exposure	Am I taking the same active substance in more than one product, or exceeding the recommended dose, dosing interval, or duration of use?	Addresses cumulative exposure, unintentional duplication, and the potential for dose-dependent toxicity.
3	Check the interaction context	Could this medicine interact with my regular medicines, other OTC products, alcohol, herbal products, or dietary supplements?	Broadens interaction assessment beyond medicine–medicine combinations and prompts consideration of relevant concomitant exposures.
4	Consider individual susceptibility	Could anything about me make this medicine less safe?	Prompts consideration of patient-specific factors that may modify contraindications, exposure, pharmacological response, or susceptibility to adverse effects.
5	Review benefit and harm	Is the medicine helping as expected, and am I experiencing unwanted or unexpected effects?	Links continued exposure to therapeutic response, tolerability, and the occurrence of possible adverse effects.
6	Stop self-treatment or seek advice	Are symptoms persistent, worsening, recurring, severe, or otherwise concerning?	Defines the boundary beyond which continued unsupervised self-treatment may be inappropriate.

The checklist is intended as a structured prompt, not as an expectation that consumers should independently resolve complex pharmacological questions. In particular, uncertainty regarding potential interactions, individual vulnerability, adverse effects, or persistent symptoms should itself prompt consultation with a pharmacist or physician. The framework is not intended to replace professional medication review, clinical assessment, or product-specific information provided in the package leaflet.

Implications for practice

The practical implication is not that consumers should perform a complete medication review before each OTC purchase. This is particularly relevant in Romania, where pharmacists have identified public education as important for safer self-medication, while patient and pharmacist reports indicate that counselling about adverse effects may not be perceived by patients as consistently as pharmacists believe it is delivered [15]. Rather, medication-safety communication should make the relevant pharmacological decision explicit. Ingredient checking should be linked to the possibility of duplicate active substances and cumulative exposure across differently branded products. Interaction checking should explicitly include prescription medicines, other OTC products, alcohol, herbal medicines, and dietary supplements. Likewise, advice regarding treatment duration should be linked not only to a specified number of days, but also to persistent, worsening, recurrent, or severe symptoms, adverse effects, and uncertainty regarding the underlying condition.

The proposed checklist provides a concise structure through which these principles could be incorporated into pharmacist counselling, patient education, pharmacy-based health-promotion initiatives, or future digital interventions. In these settings, the checklist could be used as a structured prompt to guide discussion of active substances, cumulative exposure, interaction context, patient-specific factors, therapeutic response, and the point at which self-treatment should stop and professional advice should be sought. Its purpose would be to convert broad instructions such as “check the ingredients” or “check for interactions” into explicit pharmacological questions that may identify a safety-relevant issue and, when necessary, prompt professional consultation.

The checklist has not been validated as a screening or decision-support tool, and the present study provides no evidence that its use improves medication-use behaviour or clinical outcomes. Its content validity, readability, feasibility, acceptability, and effects on risk recognition and medication-related decisions therefore require prospective evaluation before formal implementation.

Strengths and limitations

The main strength of this analysis is the respondent-level comparison of conceptually matched safety behaviours and pharmacological risk-recognition scenarios. This approach examines whether individuals reporting a recommended practice also recognise the corresponding hazard, rather than considering knowledge and behaviour as independent aggregate domains.

Several limitations should be acknowledged. This was an exploratory secondary analysis of a cross-sectional sample, and the knowledge-behaviour pairings were not developed as validated paired measures in the original questionnaire. Given the cross-sectional design, the observed associations cannot establish that reported safety behaviour causes better pharmacological knowledge or risk recognition. Behaviour was self-reported and may be affected by recall or social-desirability bias, while responses to hypothetical scenarios cannot establish actual medication-use behaviour. Dichotomisation of behavioural responses also reduced information. After correction for multiple comparisons, only the active-substance duplication association remained statistically significant; the remaining findings should therefore be interpreted primarily as descriptive and hypothesis-generating. In addition, the antihistamine-alcohol scenario did not distinguish agents according to sedative potential.

Finally, the proposed checklist is a hypothesis-generating clinical-pharmacology translation of the findings rather than a validated clinical instrument. Active-substance identification, interaction context, and the boundary of self-treatment are directly informed by the present analysis, whereas total exposure, patient susceptibility, and therapeutic response represent clinically informed extensions requiring prospective evaluation.

## Conclusions

Among adults without healthcare training, reporting recommended OTC safety behaviours did not consistently correspond to recognition of the pharmacological risks those behaviours were intended to prevent. Correspondence was strongest for active-substance duplication and more heterogeneous for interaction-related risks, while recognition of the boundary of self-treatment showed relatively low descriptive discordance.

These findings support moving beyond generic instructions such as “check ingredients” or “check interactions” toward explicit pharmacological decision points. The proposed checklist - active substance, total exposure, interaction context, patient-specific susceptibility, therapeutic response, and stop-or-escalate - provides a data-informed clinical-pharmacology framework for future OTC safety communication and intervention development, while requiring prospective evaluation before formal implementation.
